# Antibacterial Activity of *Rosmarinus officinalis, Zingiber officinale, Citrus aurantium bergamia*, *and Copaifera officinalis* Alone and in Combination with Calcium Hydroxide against *Enterococcus faecalis*

**DOI:** 10.1155/2019/8129439

**Published:** 2019-12-12

**Authors:** Silmara Silva, Nayane Alves, Priscila Silva, Thalita Vieira, Panmella Maciel, Lúcio Roberto Castellano, Paulo Bonan, Christianne Velozo, Diana Albuquerque

**Affiliations:** ^1^Department of Operative Dentistry and Endodontics, Dental College of Pernambuco, University of Pernambuco (UPE), Camaragibe, PE 54756-220, Brazil; ^2^Department of Clinical and Social Dentistry, Federal University of Paraíba (UFPB), João Pessoa, PB 58033-455, Brazil; ^3^Human Immunology Research and Education Group‐GEPIH, Health Technical School, Federal University of Paraiba, João Pessoa, PB 58033-455, Brazil

## Abstract

This study aimed to evaluate the efficacy of different concentrations of essential oils combined with calcium hydroxide against *Enterococcus faecalis*. Thirteen experimental groups were formed: NC (negative control); PC (positive control); GC (growth control); SC (sterilization control); RO (*Rosmarinus officinalis*); ROH (calcium hydroxide + RO); ZO (*Zingiber officinale*); ZOH (calcium hydroxide + ZO); AB (*Citrus aurantium bergamia*); ABH (calcium hydroxide + AB); CO (*Copaifera officinalis*); COH (calcium hydroxide + CO); DWH (calcium hydroxide and distilled water). After reconstitution of the *E. faecalis* strain, microdilution testing was performed to define the minimum inhibitory concentration (MIC) and minimum bactericidal concentration (MBC). The data were tabulated in an Excel spreadsheet, and the MIC and MBC were calculated in accordance with the Bacteriological Analytical Handbook. MICs in the range of 0 to 100 mg/ml were only observed in the ROH group. The RO, ROH, AB, ZO, and ZOH presented absolute data for MBC. Bacterial growth was detected in the DWH group at all concentrations tested. The combination of the essential oils tested here with calcium hydroxide appears promising as an intracanal medication in endodontic treatment because of its effectiveness against *Enterococcus faecalis*. Essential oils are important in endodontic therapy since calcium hydroxide, the gold standard intracanal medication, is not effective against *E. faecalis*.

## 1. Introduction

The control of *Enterococcus faecalis* is of paramount importance since this bacterium can cause bacteremia, endocarditis, intra-abdominal and pelvic infections, urinary tract infections, skin and skin structure infections, and, less frequently, central nervous system infections [1]. Its mechanisms of resistance are associated with physiological or structural changes in the bacterial cell, which is a strategy to survive the constant attack by antimicrobial agents. *Enterococcus* spp. have acquired genetic determinants that confer resistance to different classes of antibiotics [2].

In dentistry, *E. faecalis* is particularly prevalent in root canals with a diagnosis of apical periodontitis and has been implicated as the main pathogen in secondary endodontic infections [3]. In endodontic treatment, intracanal medications are used as adjuvants during biomechanical preparation. As they remain inside the root canal, these medications help eliminate surviving bacteria, prevent the proliferation of microorganisms between treatment sessions, and act as a physical-chemical barrier, preventing reinfection of the root canal and the supply of nutrients to surviving bacteria [4]. A commonly used intracanal medication is calcium hydroxide which, due to its high pH, destroys and alters the polysaccharides present in the bacterial cell wall. However, the high pH promoted by calcium hydroxide does not eliminate *E. faecalis* because this microorganism possesses an adaptive response to alkaline pH in which a proton pump carries protons into the bacterial cell to acidify its cytoplasm [5, 6].

Several phytotherapeutic compounds have been studied in pure form or combined with calcium hydroxide in order to potentiate the effects of the latter, improving the prognosis of endodontic treatment [7–11]. Plant extracts such as the essential oils of rosemary (*Rosmarinus officinalis* L.), ginger (*Zingiber officinale*), bergamot (*Citrus aurantium bergamia*), and copaiba (*Copaifera officinalis*) have been shown to possess antimicrobial, anti-inflammatory, antifungal, analgesic, antioxidant, and healing properties [8, 12, 13].

In light of the above considerations, the present study aimed to evaluate the in vitro antimicrobial activity of different concentrations of the diluted essential oils of *Rosmarinus officinalis* L., *Zingiber officinale*, *Citrus aurantium bergamia*, and *Copaifera officinalis* alone and combined with calcium hydroxide against *Enterococcus faecalis*.

## 2. Materials and Methods

### 2.1. Bacterial Strain

The standard reference strain of *Enterococcus faecalis* used in this study was obtained from the American Type Culture Collection (ATCC 14506) in the Cell Culture and Analysis Laboratory (LACEC in the Portuguese acronym), Escola Técnica de Saúde, Universidade Federal da Paraíba (ETS/UFPB). The broth microdilution method was applied to determine the minimum inhibitory concentration (MIC) and the minimum bactericidal concentration (MBC) according to the Clinical and Laboratory Standards Institute (CLSI) [14].

### 2.2. Experimental Groups

Calcium hydroxide (Biodinâmica Química e Farmacêutica Ltda., Ibiporã, PR, Brazil) was analyzed since it is the most commonly used intracanal medication in endodontic practice [5]. The essential oils and plants used in the study were high quality, 100% pure, and natural (Ferquima, Vargem Grande Paulista, SP, Brazil). The composition of the essential oils is shown in Table 1.

A pilot study was conducted for the dilution of calcium hydroxide in the essential oils. The dilutions were prepared in 6.7% Tween 80® (Sigma P4780, Steinheim, Germany), which acted as a ligand between calcium hydroxide and the essential oils, and 16% distilled water. The amount of calcium hydroxide used followed the proportions established for Calen paste (S.S. White Duflex, Rio de Janeiro, Brazil), which contains 49.77 g% of calcium hydroxide. The groups were divided as described in Table 2.

The use of control groups was necessary to evaluate the feasibility of the study. Tween 80 was tested to rule out the possibility of any activity against *E. faecalis*. Chlorhexidine gel was used based on its activity against aerobic and anaerobic Gram-positive and Gram-negative microorganisms and yeasts [15].

### 2.3. Determination of Minimum Inhibitory and Minimum Bactericidal Concentrations

The *E. faecalis* strain was reconstituted, inoculated into glass test tubes (12 mm in diameter × 75 mm in length; TWA, Biosystems) containing 3 ml brain heart infusion (BHI) broth (HiMedia Laboratories Pvt Ltd., L.B.S. Marg, Mumbai, India), and incubated in a B.O.D. incubator (MA 415/S) for 24 h in a 5% CO_2_ atmosphere at 37°C. After this period, the microorganisms were subcultured in sterile glass Petri dishes (90 × 15 mm) containing BHI agar (HiMedia) to ensure their purity and viability.

The bacterial suspensions were prepared at a concentration of 2.5 × 10^3^ CFU, inoculated into test tubes containing 5 ml of 0.9% sterile saline (0.145 mol/l NaCl), and homogenized in a vortex mixer (model MA 162) for 15 seconds. The bacterial density was standardized in a spectrophotometer (GloMax® Multi) emitting a wavelength of 625 nm to an absorbance ranging from 0.08 to 0.10, corresponding to a concentration of 1 to 2 × 10^8^ CFU/ml (0.5 McFarland standard). A 96-well flat-bottom plate (Costar®) was used for the reading, in which 100 *μ*l saline was added to the first well and 100 *μ*l of the previously prepared inoculum to the second well [14].

The MIC was determined by the broth microdilution technique [14]. First, 100 *μ*l of BHI broth was added to the U-shaped wells of a microdilution plate. Next, 100 *μ*l of the test substance was added to the first well and diluted serially by transferring a 100 *μ*l aliquot from the most concentrated well to the next well and so forth until the last well. One-hundred microliter of the inoculum (5 × 10^5^ CFU/ml) was then added to each well [14]. The assay was carried out in triplicate, and the microdilution plates were incubated under anaerobic conditions for 24 h in a 5% CO_2_ atmosphere at 37°C.

The MIC was defined as the lowest concentration of each substance that was able to inhibit bacterial growth [16]. For confirmation of the visual result, i.e., the presence of viable microorganisms at the inhibitory concentrations, 30 *μ*l of resazurin sodium dye (Sigma®) was added as a marker of oxidoreduction, which indicates a pH change due to the presence of microorganisms [17]. Each well was evaluated 1 h after application of the dye and the results were tabulated.

The MBC of the test substances and of the positive control was determined from the MIC result. For this purpose, 10 *μ*l of the corresponding inhibitory concentration and the immediately higher concentrations (MIC × 2 and MIC × 4) [18] were subcultured on Petri dishes containing BHI agar. After 24 h of incubation under anaerobic conditions, the MBC was determined using the ImageJ software and was defined as the lowest concentration that inhibited visible growth of the subculture [16].

### 2.4. Data Analysis

The data were tabulated in an Excel spreadsheet, and the MIC and MBC were calculated according to the Bacteriological Analytic Handbook [19]. Descriptive qualitative analysis of the results was performed.

## 3. Results

**Figure 1:**
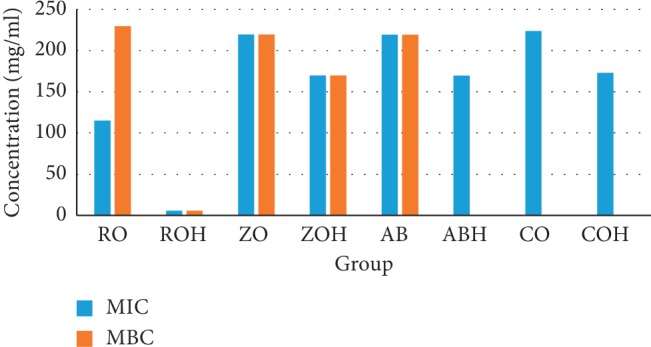
Minimum inhibitory concentration and minimum bactericidal concentration of the pure essential oils and combined with calcium hydroxide against *Enterococcus faecalis*. ^*∗*^Absolute MBC values could not be found for groups ABH, CO, and COH.

**Figure 2:**
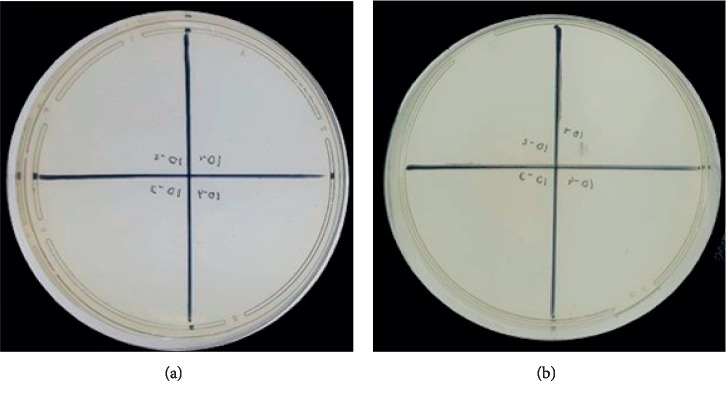
Minimum bactericidal concentration of RO (a) and ROH (b) against *Enterococcus faecalis* ATCC 14506 evaluated after 24 h of subculture on BHI agar.

The essential oils used in the research came from different regions, such as Morocco, China, Italy, and Brazil. All essential oils have been shown to possess antimicrobial, anti-inflammatory, antifungal, analgesic, antioxidant, and healing properties [8, 12, 13]. The MIC and MBC obtained for the 13 experimental groups are shown in Table 3 and in Figure 1. The MIC of the essential oils ranged from 5.5 to 223.75 mg/ml. MICs in the range of 0 to 100 mg/ml were only observed in the ROH group (Figure 2), standing out from the other groups. The RO, ROH, AB, ZO, and ZOH presented absolute data for MBC, i.e., there was no bacterial growth on the plates. The groups that did not identify MBC values were the groups in which there was no inhibition of bacterial growth at any concentration. The MIC was defined as the lowest concentration of each substance that was able to inhibit bacterial growth [16], i e., as lower the concentration is obtained, less product is needed to inhibit the bacterial growth.

The presence of Tween 80® in the dilutions had no impact on the antibacterial activity of the essential oils. There was no inhibition of bacterial growth in the negative control group.

**Figure 3:**
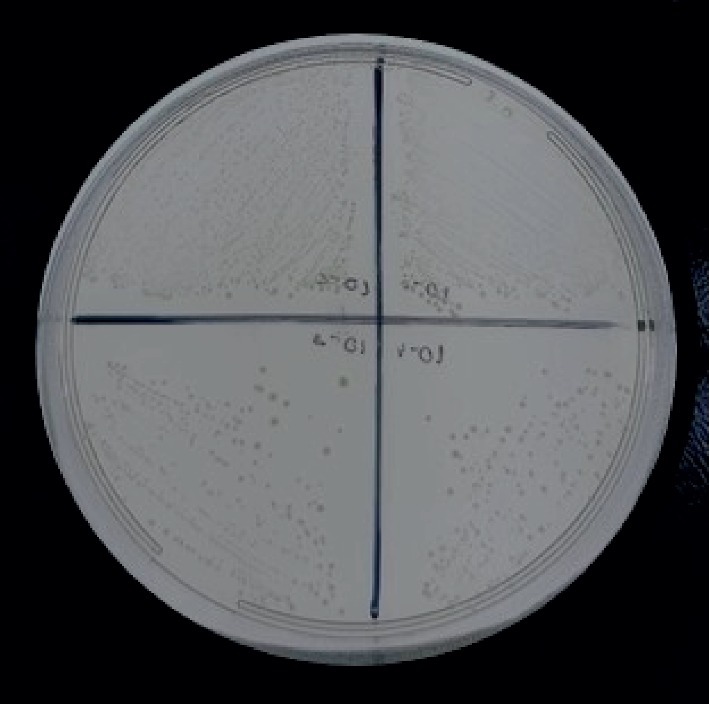
Minimum bactericidal concentration of calcium hydroxide group against *Enterococcus faecalis* ATCC 14506 evaluated after 24 h of subculture on BHI agar.

The MIC and MBC of the calcium hydroxide group could not be determined because bacterial growth was not inhibited at any of the concentrations (Figure 3).

## 4. Discussion

Various plant-derived compounds have attracted increasing attention from researchers in recent years. Natural products continue to be an important source of drugs since they are better tolerated than some synthetic compounds. Natural antimicrobial agents play an important role in different types of control of microorganisms [20]. In endodontics, calcium hydroxide is the most commonly used intracanal medication because of its high pH, which alters and destroys the polysaccharides present in the bacterial cell wall. However, this compound is ineffective against *E. faecalis* as demonstrated in the literature [21] and in the present study.

Nevares et al. [22] showed that the amount of calcium hydroxide present in an intracanal medication commonly used in endodontics was approximately twice as high as informed by the manufacturer. According to the authors, these medications should be used with caution and extrusion to periradicular tissues should be avoided because of their cytotoxicity (pH of about 12). Thus, aiming to develop more active and natural intracanal medications, essential oils are an interesting option as they contain compounds that exert antimicrobial activity, such as terpenes (monoterpenes and sesquiterpenes), terpenoids (isoprenoids), and aliphatic and aromatic compounds (aldehydes and phenols) [23]. All of these compounds are characterized by a low-molecular weight and high biocompatibility [24].

The present study showed that calcium hydroxide, at a proportion of 49.77 g% diluted in sterile distilled water, was not effective against *E. faecalis* at any of the concentrations obtained after serial dilution. The same was observed by Maekawa et al. [8] and Sirén et al. [21]; calcium hydroxide also did not exhibit activity against other microorganisms prevalent in secondary infections of the root canal system, such as *Candida albicans*. These results bring a negative perspective to the prognosis of endodontically treated teeth, especially in cases of chronic apical periodontitis due to secondary infections because they are frequently associated with the presence of *E. faecalis* [25]. Few studies in the literature have evaluated the combined use of phytotherapeutic agents and calcium hydroxide in dental applications [7–11].

As for essential oils, Ferreira Filho et al. [26] used hydroalcoholic tinctures of rosemary obtained from a compounding pharmacy and diluted in 70% alcohol at proportions ranging from 1 : 1 (pure form) to 1 : 64 and found a MIC of 14.06 mg/ml. Oliveira et al. [27] also studied commercial extracts at a concentration of 200 mg/ml propylene glycol and obtained a MIC of 50 mg/ml against *E. faecalis*. Bernades et al. [28], evaluating the essential oil of rosemary and its main pure compounds found that the latter were more active than the essential oil. Among the microorganisms tested, the pathogen *Streptococcus mitis* was the most susceptible and *E. faecalis* was the most resistant to the samples evaluated.

On the other hand, the present study demonstrated MICs of rosemary essential oil alone or combined with calcium hydroxide of 114.85 and 5.55 mg/ml, respectively. In addition, the rosemary oil exhibited better antimicrobial activity against *E. faecalis* when it was combined with calcium hydroxide. These differences in MICs between the present study and the cited studies may be related to the different methods used, such as extracts or tinctures. Essential oils, extracts, and tinctures have been shown to differ widely in their antimicrobial and antioxidant activities [23]. These divergences are associated with chemical composition and with variation within species.

Our study also showed that bergamot was effective against *E. faecalis*, with MICs of 219.25 mg/ml for the pure essential oil and of 169.5 mg/ml for the combination with calcium hydroxide. The few studies on the antimicrobial activity of bergamot were unable to demonstrate effectiveness against microorganisms such as *E. coli* [29]. Mixtures of different essential oils were also tested, but they were only effective at a very high concentration [30].

About the extract of ginger in the study by Giriraju and Yunus [31] using the agar disc diffusion test, a 10% ethanolic extract exhibited good antimicrobial potential against *Streptococcus mutans*, *E. faecalis*, and *C. albicans*, with inhibition zones of 8, 14, and 11 mm, respectively. However, the authors concluded that further studies are necessary to evaluate the practical and economic feasibility of this extract and to recommend its use in clinical practice. The present results confirm the antimicrobial potential of ginger against *E. faecalis*, with MIC and MBC of 219.5 mg/ml for the pure essential oil and of 169.75 for the oil combined with calcium hydroxide. Another study found an almost 100% microbial reduction after preparation with 20% ginger glycolic extract as auxiliary chemical substance, demonstrating high effectiveness against *C. albicans*, *E. faecalis*, and *E. coli* [9].

The oil of copaiba is brownish-yellow and has been shown to have anti-inflammatory, curative, analgesic, antibacterial, antifungal, and antitumor properties [32]. In the present study, copaiba only exhibited bacteriostatic activity against *E. faecalis*. In addition, higher concentrations were necessary for this inhibition, 223.75 mg/ml for the pure essential oil and 173 mg/ml when combined with calcium hydroxide. In the systematic review conducted by Diefenbach et al. [33], the studies demonstrated that the antimicrobial activity of copaiba oil against oral pathogens is lower than that of chlorhexidine in most cases. In only one study did copaiba oil exert an antimicrobial effect similar to the positive control. However, the authors suggested that diterpenes are an important class of plant metabolites present in copaiba oils for the identification of new antibacterial agents. The divergences in the results might be explained by the use of different copaiba species and the type of separation of the compounds and consequent antibacterial activity.

And finally, as done in other studies, chlorhexidine was used in our study as a positive control group because of its proven effectiveness against Gram-positive and Gram-negative microorganisms [15]. Similarly, Maekawa et al. [8] observed that 1% chlorhexidine gel was more effective in eliminating *E. faecalis* when compared to the other groups tested (calcium hydroxide paste and turmeric gel).

## 5. Conclusions

The combination of the essential oils tested here with calcium hydroxide appears promising as an intracanal medication in endodontic treatment because of its effectiveness against *Enterococcus faecalis*. Our study suggests that all preparations exert bacteriostatic effects against this microorganisms. However, further in vivo studies are necessary to evaluate their clinical relevance.

## Figures and Tables

**Table 1:** Composition of the essential oils used in the study.

Oil	Main components	Density (20°C)	Origin
*Rosmarinus officinalis*	1,8-cineole (40%); alpha-pinene (13%); limonene (3%); camphor (15%); beta-pinene (7%)	0.919	Morocco
*Zingiber officinale*	Alpha-zingiberene (33%); beta-bisabolene (6%); alpha-pinene (2%); beta-sesquiphellandrene (13%)	0.878	China
*Citrus aurantium bergamia*	Linalol (15%); limonene (36%); linalyl acetate (30%); gamma-terpinene (7%); beta-terpinene (5%)	0.877	Italy
*Copaifera officinalis*	Beta-caryophyllene (51%)	0.895	Brazil

Source: Ferquima, 2018.

**Table 2:** Description of the groups and proportions of intracanal medications analyzed in the study.

Group	Substances	Dose
NC (negative control)	Tween 80®	—
PC (positive control)	Chlorhexidine (2%)	—
GC (growth control)	BHI medium + microorganism	—
SC (sterilization control)	BHI medium	—
RO	*Rosmarinus officinalis* (rosemary)	—
ROH	Calcium hydroxide	7.5 mg
	*Rosmarinus officinalis*	11.6 ml
	Tween 80®	1 ml
	Sterile distilled water	2.4 ml
ZO	*Zingiber officinale* (ginger)	—
ZOH	Calcium hydroxide	7.5 mg
	*Zingiber officinale*	11.6 ml
	Tween 80®	1 ml
	Sterile distilled water	2.4 ml
AB	*Citrus aurantium bergamia* (bergamot)	—
ABH	Calcium hydroxide	7.5 mg
	*Citrus aurantium bergamia*	11.6 ml
	Tween 80®	1 ml
	Sterile distilled water	2.4 ml
CO	*Copaifera officinalis* (copaiba)	—
COH	Calcium hydroxide	7.5 mg
	*Copaifera officinalis*	11.6 ml
	Tween 80®	1 ml
	Sterile distilled water	2.4 ml
DWH	Calcium hydroxide	7.5 mg
	Sterile distilled water	15 ml

**Table 3:** Minimum inhibitory concentration and minimum bactericidal concentration of the pure essential oils and combined with calcium hydroxide against *Enterococcus faecalis*.

Group	MIC (mg/ml)	MBC (mg/ml)
RO	114.87	229.74
ROH	5.55	5.55
ZO	219.5	219.5
ZOH	169.75	169.75
AB	219.25	219.25
ABH	169.5	ni^*∗∗*^
CO	223.75	ni^*∗∗*^
COH	173	ni^*∗∗*^
DWH	ne^*∗*^	ne^*∗*^
CP	Effective	Effective
CN	ne	ne
CE	a	a
CC	b	b

MIC = minimum inhibitory concentration; MBC = minimum bactericidal concentration; ^*∗*^ne = noneffective; ^*∗∗*^ni = not identified; a = bacteria absent; b = bacteria present.

## Data Availability

The data used to support the findings of this study are included within the article.
